# Childhood maltreatment is associated with lower exploration and disrupted prefrontal activity and connectivity during reward learning in volatile environments

**DOI:** 10.1111/jcpp.14095

**Published:** 2024-12-12

**Authors:** Diana J. N. Armbruster‐Genç, Louise Neil, Vincent Valton, Harriet Phillips, Georgia Rankin, Molly Sharp, Jessica Rapley, Essi Viding, Jonathan P. Roiser, Eamon McCrory

**Affiliations:** ^1^ Division of Psychology and Language Sciences University College London London UK; ^2^ Institute of Psychology University of Kaiserslautern‐Landau (RPTU) Landau Germany; ^3^ Institute of Cognitive Neuroscience University College London London UK

**Keywords:** Maltreatment, internalising disorder, neuroimaging, learning

## Abstract

**Background:**

Atypical reward processing is implicated in a range of psychiatric disorders associated with childhood maltreatment and may represent a latent vulnerability mechanism. In this longitudinal study, we investigated the impact of maltreatment on behavioural and neural indices of reward learning in volatile environments and examined associations with future psychopathology assessed 18 months later.

**Methods:**

Thirty‐seven children and adolescents with documented histories of maltreatment (MT group) and a carefully matched group of 32 non‐maltreated individuals (NMT group) aged 10–16 were presented with a probabilistic reinforcement learning task featuring a phase of stable and a phase of volatile reward contingencies. Brain activation and connectivity were assessed simultaneously using functional magnetic resonance imaging (fMRI). Computational models were used to extract individual estimates of learning rates and temperature, and neural signals in prespecified regions of interest were analysed during volatile and stable environments. In regression analyses, behavioural measures and neural signals at baseline were used to predict psychological symptoms at follow‐up.

**Results:**

The MT group showed lower behavioural exploration, which predicted decreased internalising symptoms at follow‐up. The MT group had lower activation in the orbitofrontal cortex (OFC) during outcome delivery in volatile relative to stable contexts. OFC connectivity with an area in the mid‐cingulate cortex was also lower during outcome processing, which predicted higher general psychopathology at follow‐up.

**Conclusions:**

These findings are consistent with the notion that low exploratory behaviour following childhood maltreatment is potentially a protective adaptation against internalising symptoms, while disrupted neural processing of reward learning in volatile environments may index latent vulnerability to mental illness.

## Introduction

Childhood maltreatment, such as neglect and abuse, is associated with long‐term risk for psychiatric disorders, including major depression and anxiety disorders (Gilbert et al., 2009). However, the neurocognitive mechanisms underlying such risk are not well understood. It has been suggested that alterations in neurocognitive functioning, including attention to threat (McCrory et al., 2013), autobiographical memory (McCrory et al., 2017), emotion regulation (McLaughlin, Peverill, Gold, Alves, & Sheridan, 2015), trust (Neil et al., 2022), reward (Goff et al., 2013) and effort processing (Armbruster‐Genç et al., 2022), arise following adaptation to early adverse environments. The concept of ‘latent vulnerability’ (McCrory & Viding, 2015) refers to the way in which such adaptations may render neurocognitive systems poorly optimised to respond to more normative environments, such as school or a more predictable home environment (e.g. foster family environment). Over time, it is thought that this mismatch contributes to an increased likelihood of poor mental health, social and educational outcomes.

From a developmental perspective, childhood environments characterised by abuse or neglect are likely to result in highly atypical reinforcement learning histories, where rewards and punishments are less predictable, less contingent and qualitatively different from those encountered during more normative caregiving contexts (Bousha & Twentyman, 1984; Cyr, Euser, Bakermans‐Kranenburg, & Van Ijzendoorn, 2010). These atypical learning histories can impact value‐based decision making in the context of reward processing and reward learning – two transdiagnostic mechanisms implicated in several psychiatric disorders commonly associated with maltreatment experience, including depression and anxiety (Browning, Behrens, Jocham, O'Reilly, & Bishop, 2015). For example, previous research has found that major depression is robustly associated with impairments in reward learning (Halahakoon et al., 2020) and blunted striatal activation during the anticipation and receipt of reward (Keren et al., 2018). In non‐clinical samples, lower striatal activation has been shown to be associated with future onset of depressive symptoms (Stringaris et al., 2015). Neural circuit models of reward processing and depression overlap (Haber & Behrens, 2014; Haber & Knutson, 2010; Keren et al., 2018) and highlight, in addition to the striatum, the central involvement of prefrontal regions, in particular the orbito‐frontal (OFC) and anterior/mid‐cingulate cortex (ACC/MCC), as well as connectivity between these regions (Admon et al., 2015). These findings suggest that alterations in reward processing may play an important role in the aetiology and maintenance of depression.

Children and adolescents with maltreatment experience show both behavioural (Dillon et al., 2009) and neural alterations during reward processing (Kasparek et al., 2023). Blunted striatal activation during reward anticipation (Dennison et al., 2016; Hanson, Hariri, & Williamson, 2015), lower ACC/MCC activation during effort‐based decision making for reward (Armbruster‐Genç et al., 2022), and blunted activation in a network including the striatum and the OFC during expected value processing have all been reported (Blair et al., 2022; Gerin et al., 2017). Longitudinal studies have found that these alterations are associated with risk for internalising symptomatology (e.g. Armbruster‐Genç et al., 2022). A recent meta‐analysis found a consistent association between childhood adversity and impaired reward processing, with the largest effect size for reward learning (Oltean, Soflau, Miu, & Szentagotai‐Tatar, 2022). Identifying the specific reward‐related processes that link maltreatment experiences with the risk of developing depression has the potential to advance our mechanistic understanding of mental health risk following early adversity, and inform our approaches to prevention and intervention.

Caregiving environments in which children experience abuse and neglect are more likely characterised by more volatile and unpredictable reinforcements. An important question concerns whether such exposure to atypical environments in the past influences how children weight recent outcomes when making decisions in the present. In relatively stable environments it is adaptive to accord less weight to recent outcomes, compared to more volatile environments in which it is more adaptive to accord greater weight to recent outcomes. Behrens, Woolrich, Walton, and Rushworth (2007) showed in a healthy adult sample that a region in the ACC/MCC is involved in signalling an environment's volatility. While previous research has identified important differences in the ways individuals with childhood maltreatment histories learn about rewards in static environments (Blair et al., 2022; Gerin et al., 2017), the impact of early adversity on the neurocognitive underpinnings of reward learning under volatility has yet to be investigated. Interestingly, previous studies have shown that individuals with adverse childhood experiences underweight reward feedback and are less exploratory (Humphreys et al., 2015; Lloyd, McKay, & Furl, 2022; Xu, Harms, Green, Wilson, & Pollak, 2023), raising the question of whether differences in volatility processing may contribute to reward learning impairments.

The aim of the present study was to investigate how maltreatment experience in childhood impacts reward learning in environments characterised by different levels of volatility. Specifically, we aimed to investigate both behavioural and neural correlates of reinforcement learning across environments characterised by stable and volatile reward statistics. We adopted a longitudinal design, recruiting a group of children and adolescents with documented maltreatment experience and a matched control group of typically developing children, to explore whether alterations in reward processing were associated with future symptoms.

At the behavioural level we predicted a poorer ability to adjust learning rates between stable and volatile environments in those with maltreatment experience (MT group). We also hypothesised that maltreatment experience would be associated with lower exploratory behaviour, as reported in previous studies (Frankenhuis & Gopnik, 2023; Humphreys et al., 2015; Lloyd et al., 2022; Xu et al., 2023). At the neural level, we predicted that a poorer ability to adjust learning rates in the MT group would be reflected in poorer tracking of environmental volatility in brain regions that have previously been shown to function atypically in individuals with maltreatment experience, including the ACC/MCC (Armbruster‐Genç et al., 2022), the OFC and the striatum (Blair et al., 2022; Gerin et al., 2017). We additionally assessed volatility‐modulated functional connectivity with brain regions that showed group differences in activation. Finally, we investigated the relationship between neural activation during reward learning and future symptomatology, with a focus on internalising symptomatology and those regions thought to function atypically in individuals with maltreatment experience; broader effects on general psychopathology were assessed in exploratory analyses.

## Methods

### Participants and ethical considerations

This study was approved by the UCL Research Ethics Committee. A total of 69 young people aged 10–16 years were recruited: 37 from London Social Services with a documented history of maltreatment (MT group), and 32 young people with no maltreatment history (NMT group). The participants' legal guardians provided written consent, and all participants provided written assent. Groups did not differ with regards to sex, age, ethnicity, socioeconomic status (SES), IQ and pubertal status (Table 1). Complete follow‐up data were available for 27 MT and 29 NMT participants.

**Table 1:** Background and psychopathology measures at baseline and follow‐up

	Baseline			Follow‐up		
	MT group	NMT group	*p*	MT	NMT group	*p*
	(*N* = 37)	(*N* = 32)		(*N* = 27)	(*N* = 29)	
Background measures						
Sex, female: *n* (%)	20 (54)	17 (53)	.938	16 (59)	16 (55)	.757
Age (*SD*)	13.7 (2.0)	13.5 (2.2)	.718	15.1 (2.1)	15.3 (2.2)	.733
Pubertal status (*SD*)	2.6 (0.8)	2.3 (0.9)	.180	2.7 (0.9)	3.0 (0.8)	.288
Ethnicity, White: *n* (%)^a^	16 (43)	15 (47)	.762	10 (37)	15 (52)	.269
Socioeconomic status (*SD*)	3.3 (1.0)	3.2 (0.8)	.523	3.1 (1.0)	3.1 (0.7)	.721
Wechsler Abbreviated Scales of Intelligence II IQ (*SD*)	100.6 (11.7)	102.1 (9.3)	.579	101.7 (11.1)	102.4 (9.6)	.798
Psychopathology measures						
SDQ total score (*SD*) (parent report)	11.2 (6.8)	6.6 (4.3)	.001*	11.9 (7.4)	8.3 (4.2)	.035
Emotional symptoms (*SD*)	2.8 (2.3)	1.5 (1.5)	.007*	3.8 (2.7)	1.9 (1.7)	.004*
Conduct problems (*SD*)	2.5 (2.4)	1.0 (1.2)	.002^b^	1.9 (2.1)	1.4 (1.3)	.241^b^
Hyperactivity (*SD*)	4.1 (2.7)	2.4 (2.3)	.006^b^	3.9 (2.8)	2.8 (2.3)	.100^b^
Peer problems (*SD*)	1.9 (1.6)	1.7 (1.8)	.657^b^	2.3 (1.7)	2.3 (1.8)	.972^b^
Prosocial behaviour (*SD*)	8.2 (1.8)	9.2 (1.2)	.006^b^	8.2 (2.0)	8.4 (1.4)	.626^b^
CASI major depressive episode (*SD*) (parent report)	6.4 (4.3)	3.5 (2.6)	.001^b^	5.5 (3.9)	4.5 (2.9)	.286^b^
CTQ Childhood Trauma Questionnaire (*SD*) (child report)	18.8 (10.8)	12.7 (2.7)	.009^b^	–	–	–

CASI, Child and Adolescent Symptom Inventory; CTQ, Childhood Trauma Questionnaire; SDQ, Strengths and Difficulties Questionnaire.

^a^ For a complete breakdown of ethnicities see Table S2.

^b^ For illustrative purposes and in order to allow comparison with previous studies in terms of group characterisation, not part of hypotheses or interpretation.

* *p* < .05 surviving correction for multiple comparison.

### Measures

Participants attended an fMRI session and young people as well as carers completed questionnaires at baseline and at follow‐up (follow‐up interval on average 19.1 ± 1 months; SI: Table S5): Caregivers completed the Strengths and Difficulties Questionnaire (SDQ; Goodman, 1997) and the Child and Adolescent Symptom Inventory (CASI; Gadow & Sprafkin, 2005). The SDQ emotional problems subscale served as measure for internalising symptoms and was the primary focus of our hypotheses. The SDQ Total Score is a standard composite score of all subscales (omitting the prosocial behaviour subscale) and was used in our additional exploratory analyses. All other SDQ subscales as well as the CASI are reported in order to allow comparison with previous studies from our group and others in terms of group characterisation (e.g. Armbruster‐Genç et al., 2022; Gao et al., 2022; Gerin et al., 2017; Pandey et al., 2020). Young participants completed the Childhood Trauma Questionnaire (CTQ; Bernstein et al., 2003) at baseline; this measure was also included to allow comparison of our sample with other studies in the field (e.g. Hanson et al., 2015). Cognitive ability was assessed using two subtests of the Wechsler Abbreviated Scales of Intelligence (WASI‐II; Wechsler, 2011); the subscales ‘Vocabulary’ and ‘Matrix Reasoning’ were administered resulting in the FSIQ‐2 score providing an estimate of general cognitive ability. Pubertal status was assessed using the Pubertal Development Scale (PDS; Petersen, Crockett, Richards, & Boxer, 1988); scores range from one ‘pre‐pubertal’ to five ‘post‐pubertal’. To integrate both self‐ and parent‐report data, means were calculated across these scores. SES was measured as the average of the carers' educational levels. At follow‐up, carers additionally completed the Coddington Life Events Scale (Coddington, 1972) (see also Supporting Information).

#### Maltreatment history

All participants in the MT group had experienced a level of maltreatment requiring social service intervention. Social workers rated (from 0 to 4) the occurrence and severity of neglect, emotional abuse, sexual abuse and home violence (physical abuse/intimate partner violence) based on social services' file information (Kaufman, Jones, Stieglitz, Vitulano, & Mannarino, 1994; Table S1).

#### Task

**Figure 1:**
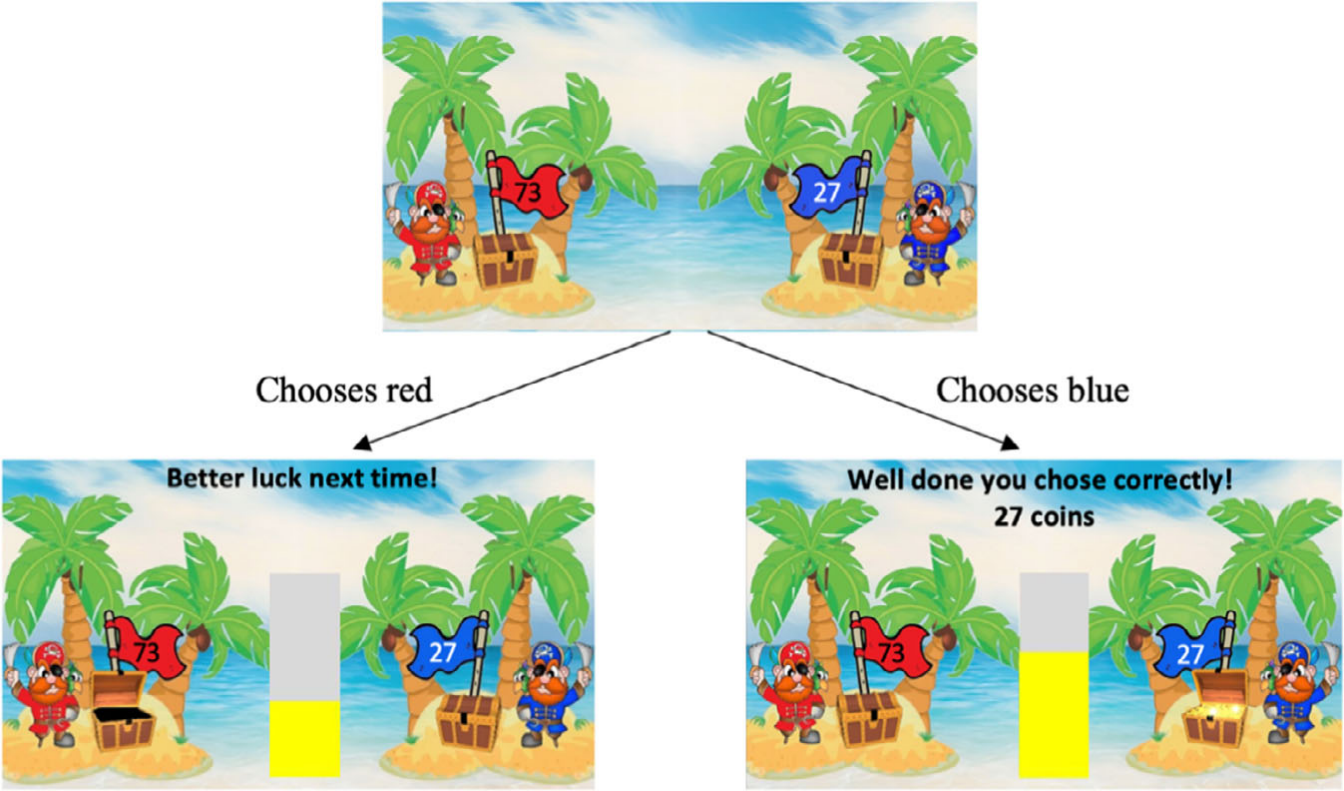
Illustration of volatility paradigm. Figure adapted from Manning et al. (2017)

Task stimuli were presented in Matlab (Mathworks Inc.) using the Cogent 2000 toolbox (Figure 1). Participants were presented with a choice between two pirates, each putting up a flag showing the varying amounts of coins that they claimed to have in their treasure chest. Participants were instructed that on each trial, only one of the pirates was telling the truth about the amount of coins in his chest, and that the other chest was actually empty. If the correct pirate was chosen, participants won the coins; there were no losses. The task included a total of 120 trials: across 60 trials reward contingencies were stable (stable phase) in that in 75% of the trials one of the pirates revealed the reward if chosen, whereas the other pirate had the reward only in 25% of the trials; across the other 60 trials, reward contingencies changed every 20 trials (volatile phase) with one of the pirates revealing the reward in 80% of the trials, the other pirate only in 20% of the trials. Starting phase (stable or volatile) was counterbalanced across participants and groups and was also included as nuisance variable in the fMRI models. For adaption for use in the fMRI scanner, trial timings were changed in line with Behrens et al. (2007).

### Data analysis

#### Computational analyseBas

A computational modelling approach was used to analyse the participants' decisions during the task on a trial‐by‐trial basis. A variety of reinforcement learning models of increasing complexity were built to capture the influence of learning rates, exploration and starting biases on behaviour. All models were implemented in a hierarchical manner, such that parameter estimates were recovered using hierarchical Bayesian estimation through the application of soft constraints on likely parameter ranges using prior distributions (Gelman & Hill, 2007; Valton, Wise, & Robinson, 2020). The Widely Applicable Information Criterion (WAIC) scale and K‐fold cross validation (K‐fold CV) were used to compare model fits (Vehtari, Gelman, & Gabry, 2017; Watanabe, 2013) to select the most parsimonious model (i.e. the model that best captured the participants' performance, while accounting for model complexity). We first performed model comparison at the participant level, comparing each successive model against the Null model. This allowed the identification of participants who did not appear to be sufficiently well captured by the 12 modelling strategies implemented. Thirty‐five percent of the participants demonstrated better evidence for the Null model, hence they were removed from further computational analysis as including them would have heavily skewed the model comparison results at the group level towards a simpler model than warranted (given that group model comparison is an equal‐weighted sum of participants model evidence). Additionally, the estimated parameters for these participants would have been uninterpretable due to their poor model fit. Importantly, the groups of MT and NMT participants remained matched on all matching variables (all *p* > .203); also, there were no significant differences across groups between those with poor model fit and those showing sufficient model fit (all *p* > .060). Here, we only present the winning model (details in Supporting Information), which comprised four parameters per participant: a starting bias that represents the participant's initial preference for one stimulus over another; a temperature parameter capturing exploration; and two learning rates (separately for the stable and volatile environments) describing how quickly task feedback affects the participants' internal representation of the two options.

#### FMRI analyses

Data were analysed using SPM12 (https://www.fil.ion.ucl.ac.uk/spm/software/spm12) implemented in Matlab (MathWorks Inc.). Details about fMRI data acquisition, preprocessing and subject level analysis can be found in the Supporting Information. Based on previous research, we defined ROIs anatomically for the striatum, the ACC and neighbouring MCC (extracted from the AAL atlas; Rolls, Huang, Lin, Feng, & Joliot, 2020), as well as the OFC (extracted from the Brodmann atlas in MRIcron; Rorden, Karnath, & Bonilha, 2007). Results were family wise error (FWE) small‐volume corrected in SPM12. ROI activation for the following contrasts was compared between groups in *model 1* (computationally derived regressors) for the parametric modulator ‘expected value’ in the volatile versus stable phase, as well as for the parametric modulator ‘prediction error’ in the volatile versus stable phase. In *model 2* (model agnostic), group‐wise comparisons were conducted for the main effect of volatile versus stable phase during the decision and the outcome phase (i.e. averaging across win/no‐win trials) as well as their interactions with win/no‐win. We also conducted exploratory whole‐brain analyses (*p* < .05 FWE corrected, details see Supporting Information) for these contrasts. All second‐level analyses included experimental order (stable/volatile phase first), age, sex and pubertal status as covariates of no interest.

Functional connectivity within the network of ROIs was further explored: we conducted a PPI analysis in SPM12 with a left orbitofrontal cluster that differed between the groups [−18 + 42–8] (see “Results” section) as a seed region. The psychological regressor was observing outcomes during the volatile versus stable phase, and the PPI was computed in SPM12 using a deconvolution approach.

#### Longitudinal analyses

Prediction of our primary measure of interest, internalising symptomatology (SDQ emotional problems), was performed including the following regressors: baseline symptomatology, sex, age, IQ, pubertal status, neurocognitive measures of interest (i.e. those showing a significant group difference at baseline – behavioural: temperature; neural: OFC activation and OFC functional connectivity), group and interactions between group and the neurocognitive measures of interest. To complement this hypothesis‐driven analysis, we also explored prediction of general psychopathology (SDQ Total).

#### Power analysis for observed data

Given the sample size included in our analyses (*N* = 37 MT, *N* = 32 NMT), we calculated, using the G*Power (Faul, Erdfelder, Lang, & Buchner, 2007) ‘sensitivity’ option, that we had at least 80% power to detect group differences of *d* > 0.70 at α = 0.05 (two‐tailed). This is comparable to effect sizes reported in previous cross‐sectional (Gerin et al., 2017) and longitudinal work (Dennison et al., 2016).

## Results

As expected, the MT group reported higher levels of internalising symptoms on the SDQ emotional problems subscale both at baseline and follow‐up (Table 1). The MT group also presented with significantly elevated general psychopathology indexed by the baseline SDQ total score. As described before, we focused on internalising symptomatology with exploratory analyses targeting general psychopathology. In the total sample, for 81% of participants complete follow‐up data were available. Retention rates did not differ significantly between groups (MT: 73% retained, NMT: 91%; *Χ* ^2^(1) = 3.50, *p* = .061). The follow‐up interval did not differ significantly between groups (Table S5). In the MT group, retained participants had higher socioeconomic status than dropped out participants (*Mann–Whitney‐U* = 55.5, *p* = .005). There were no significant differences between retained and dropped‐out participants in the MT or the NMT group in terms of maltreatment severity, baseline emotional and general symptomatology, age, sex, IQ or pubertal status (all *p* > .109; Table S6).

### Behavioural results

**Figure 2:**
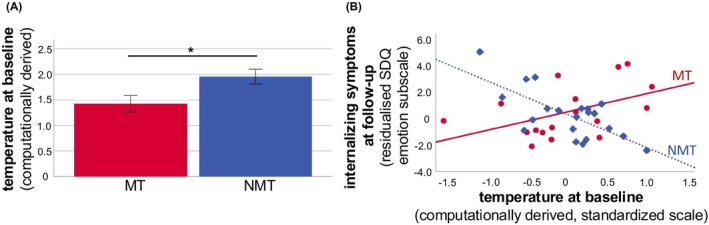
Temperature group difference and partial regression plot. (A) Significant difference between MT and NMT group in the computationally derived temperature parameter indicating less exploratory behaviour in MT participants. Error bars show ±1 SE. (B). Partial regressions predicting follow‐up internalising symptoms from baseline temperature parameter in each group, *y*‐axes shows residual scores, *x*‐axis scores were standardised. The regression slopes differed significantly; MT group: red line and dots; NMT group: blue broken line and diamond shapes. *p < .05

The groups did not differ in the total number of coins collected (*t*(67) = 0.71, *p* = .483). Examining group differences in the computationally derived behavioural measures (for those participants with adequate model fit: *N* = 24 MT, *N* = 22 NMT; see “Methods” section and Supporting Information), we found that the MT group displayed significantly lower temperature values (*t*(44) = 2.40, *p* = .021), indicating less exploratory behaviour (Figure 2A). As expected, learning rates (LR) were significantly higher in the volatile phase across both groups (LR_stable_: mean = 0.35, *SD* = 0.07; LR_volatile_: mean = 0.41, *SD* = 0.17; *t*(45) = −2.7, *p* = .009). There were, however, no significant differences between groups (LR_stable_ MT group: mean = 0.36, *SD* = 0.08; LR_stable_ NMT group: mean = 0.33, *SD* = 0.04; *t*(44) = 1.54, *p* = .13; LR_volatile_ MT group: mean = 0.41, *SD* = 0.19; LR_volatile_ NMT group: mean = 0.42, *SD* = 0.14; *t*(44) = 0.24, *p* = .81).

Analyses of longitudinal data indicated that at follow‐up, internalising symptoms were significantly predicted by baseline temperature values, but in opposite directions in the two groups (significant temperature‐by‐group interaction, controlling for baseline emotional symptoms, sex, age, IQ and pubertal status): β = −.80, *t*(37) = −4.03, *p* < .001; ${r^{2}}_{\text{part}}$ = −.49 (total model: *R* ^2^ = .58, *F*(8,37) = 18.1, *p* = .001). Tests of simple effects, that is separate regression analyses within each group showed a significant negative association between exploratory behaviour and internalising symptoms in the NMT group (β = −.73, *t*(20) = −3.66, *p* = .003; ${r^{2}}_{\text{part}}$ = −.60; total model: *R* ^2^ = .63, *F*(6,14) = 3.95, *p* = .016), while this was not the case in the MT group (β = .43, *t*(16) = 1.72, *p* = .116; ${r^{2}}_{\text{part}}$ = .36; total model: *R* ^2^ = .57, *F*(6,10) = 2.22, *p* = .127; Figure 2B). The exploratory analysis regarding general psychopathology was not significant (controlling for baseline emotional symptoms, sex, age, IQ and pubertal status): β = −.22, *t*(37) = −1.02, *p* = .318; ${r^{2}}_{\text{part}}$ = −.14 (total model: *R* ^2^ = .505, *F*(8,37) = 3.57, *p* = .006). Further results of exploratory analyses regarding externalising symptoms can be found in the Supporting Information (Table S4).

### FMRI results

**Figure 3:**
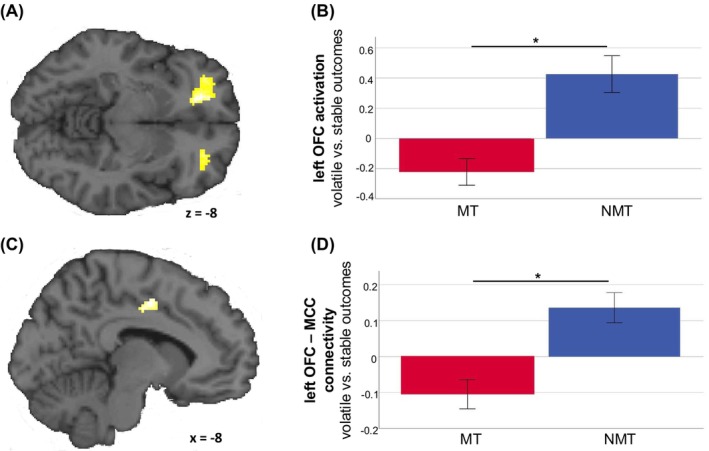
Significant activation and connectivity differences between groups in the left OFC. (A) Left OFC cluster (*p* < .001 (uncorrected), *k* = 100 for illustrative purposes). (B) Compared to the NMT group the MT group had lower activation to outcomes during the volatile phase (vs. stable phase). Error bars show ±1 SE. (C) Cluster in the mid‐cingulate cortex (MCC) showing significant connectivity differences between groups (*p* < .001, *k* = 100 for illustrative purposes) from the psychophysiological interaction analysis (PPI). (D) Left OFC–MCC PPI: compared to the NMT group the MT group had lower functional connectivity during outcomes in the volatile condition compared to the stable condition. Error bars show ±1 SE. *p < .05 FWE corrected

No significant group differences were observed in the computationally informed fMRI analysis (*model 1*), either in the ROI analyses or across the whole brain. However, additional results across the entire sample provided evidence that computational reinforcement learning processes, for example prediction error, do map on to the expected neural substrates in this sample, including the striatum (see Figure S2). Results from the typical non‐computational analysis (*model 2*) showed a significant group difference in a predefined ROI, the left orbitofrontal cortex (OFC; peak [−18 + 42–8]; *Z* = 4.48, *p* < .05 SV‐FWE corrected) for the main effect of volatile versus stable during the outcome phase. The MT group showed lower activation in this left OFC cluster during the volatile phase compared to the NMT group (Figure 3A,B). No other group differences were observed in our other ROIs (the ACC/MCC and striatum) or in the whole‐brain analyses. Exploratory analyses did not identify significant associations between OFC activation and internalising symptoms (MT: *r* = −.08, *p* = .637; NMT: *r* = −.27, *p* = .143) or general symptomatology symptoms (MT: *r* = .08, *p* = .638; NMT: *r* = −.22, *p* = .242) within groups. There was a negative correlation between OFC activation and internalising symptomatology across groups which, however, did not survive correction for multiple comparison (*r* = −.28, *p* = .020). There was no correlation between OFC activation and general symptomatology across groups (*r* = −.21, *p* = .089). Longitudinal analyses did not show any significant prediction of internalising symptoms (β = −.23, *t*(54) = −1.21, *p* = .234; ${r^{2}}_{\text{part}}$ = −.13; total model: *p* = .455, *F*(8,46) = 4.90, *p* < .001) or general symptomatology (β = −.15, *t*(54) = −1.27, *p* = .210; ${r^{2}}_{\text{part}}$ = −.13; total model: *R* ^2^ = .558, *F*(8,46) = 7.25, *p* < .001) by left OFC activation (controlling for baseline emotional symptoms, sex, age, IQ and pubertal status). Further results of exploratory analyses regarding externalising symptoms can be found in the Supporting Information (Table S4).

The PPI analysis with the OFC cluster as seed region showed lower connectivity to a cluster in the MCC (peak coordinate: [−8–4 + 46]; *Z* = 4.18, *p* < .05 SV‐FWE corrected) for outcomes in the volatile (relative to the stable) condition in the MT group, compared to the NMT group (Figure 3C,D). To explore this result further, we analysed the correlation between left OFC‐MFC connectivity and behavioural temperature, which was non‐significant, both across groups (*r* = .114, *p* = .450) and within groups (MT: *r* = −.083, *p* = .701; NMT: *r* = .007, *p* = .974). Additional exploratory whole brain analyses of OFC connectivity did not show any further significant results.

Finally, longitudinal analyses predicting internalising symptomatology at follow‐up from OFC‐MCC PPI were non‐significant (controlling for baseline emotional symptoms, sex, age, IQ and pubertal status): β = .20, *t*(54) = 1.21, *p* = .234; ${r^{2}}_{\text{part}}$ = .13 (total model: *R* ^2^ = .455, *F*(8,46) = 4.90, *p* < .001). Additional exploratory analyses predicting general symptomatology showed an interaction with group (controlling for baseline general symptoms, sex, age, PDS and IQ): β = .30, *t*(54) = 2.01, *p =* .05; ${r^{2}}_{\text{part}}$ = .29 (total model: *R* ^2^ = .57, *F*(8,46) = 7.7, *p* < .001), but this did not survive correction for multiple comparisons.

## Discussion

We investigated whether volatility of the reward environment is processed differently in children and adolescents with maltreatment experience. Consistent with our hypotheses, these individuals showed less exploratory behaviour than their non‐maltreated peers, a pattern that in turn predicted lower internalising symptomatology longitudinally. Maltreated individuals also had atypical neural reward processing when in a volatile environment, specifically lower OFC activation during outcome presentation. Children and adolescents with maltreatment experience also showed lower OFC‐MCC connectivity during outcome presentation in the context of high volatility. However, contrary to our hypothesis maltreatment experience was not associated with differences in learning rates.

In the current study, we found that children and adolescents with maltreatment experience made more deterministic decisions, a corollary of lower exploratory propensity which is consistent with previous research in adult (Lloyd et al., 2022) and adolescent samples (Humphreys et al., 2015; Xu et al., 2023). In some contexts this can lead to suboptimal reward outcomes – for example when reward contingencies shift (Cohen, McClure, & Yu, 2007). It has been suggested that such a tendency to prefer options with known outcomes could be adaptive in adverse and unstable environments (Cohen et al., 2007) where the consequences might be less certain and perhaps associated with a greater risk of punishment. Lower exploratory behaviour can also be conceptualised from an attachment perspective, where establishing a secure attachment base is postulated to represent a prerequisite to supporting exploration (Bowlby, 2008); importantly, experience of childhood maltreatment is associated with an increased likelihood of developing a non‐secure attachment style (Baer & Martinez, 2006). In typically developing children, we found a significant association between higher exploratory behaviour and lower future internalising symptoms at follow‐up. This is consistent with research showing generally increased exploration in children and adolescents and the notion that this represents an adaptive learning mechanism in normative development (e.g. Schulz, Wu, Ruggeri, & Meder, 2019). However, in the MT group the association between exploratory behaviour and lower internalising symptoms was non‐significant (and in the opposite direction), suggesting that this adaptive learning mechanism may be less relevant following childhood maltreatment. We speculate that this may represent an adaptation which provides short‐term advantages (reduced exposure to situations that may be anxiety provoking) but that potentially incurs long‐term costs, including reduced opportunities to identify new sources of reward. For example, it may reduce the likelihood of establishing new social connections, a pattern that may compound over time, potentially contributing to social thinning (McCrory, Foulkes, & Viding, 2022). Early environments characterised by the absence of reward might also affect the development of reward sensitivity – a pattern that has been identified before in extreme cases of deprivation (Goff et al., 2013). Drawing on computational literature, cognitive science and evolutionary theories, it has recently been argued that lower exploration behaviour after early adversity might represent early maturation, that is an earlier shift from exploration towards exploitation, as an adaptation to adverse environments (Frankenhuis & Gopnik, 2023). Longer term follow‐up beyond the 18 months investigated here is needed to investigate these hypotheses further.

Previous research has consistently shown that the OFC is involved in the processing of reward outcomes in both humans and non‐human primates (Bechara, 2000; Kringelbach & Rolls, 2004). Across studies, evidence suggests the involvement of different subregions of the OFC in the processing of different kinds of reward values (e.g. sensory, monetary) as well as in the processing of reward and punishment (Kringelbach & Rolls, 2004). Several studies have shown a pattern of hypoactivation of prefrontal regions (including the OFC) during reward outcome processing in association with depressive disorder (Dichter, Kozink, McClernon, & Smoski, 2012; McCabe, Woffindale, Harmer, & Cowen, 2012). In the present study, we found that the OFC was less activated in individuals with maltreatment experience during the processing of both negative and positive outcomes, specifically in a volatile reward environment. In signalling incentives as well as punishments, the OFC is likely to influence future decision making (Deco & Rolls, 2006) such that a hypoactivation of the OFC can result in changes in goal‐directed behaviours which, in turn, might set the individual on a trajectory towards depressive disorder. Taken together, these results indicate that after childhood maltreatment especially volatile reward conditions affect the representation of reward value in the OFC, for example by reducing the affective value a stimulus is associated with. This might represent a latent vulnerability to depression.

We also found lower functional connectivity between the OFC and an area in the MCC during the processing of reward outcomes under volatility in maltreated individuals. A similar region has been implicated in general reward‐based decision making in a study using the same paradigm of reinforcement learning under volatile conditions in adults (Behrens et al., 2007). This finding is again consistent with the notion that maltreatment experience is associated with altered processing of reward value. Such functional alterations in the neural processing of reward after childhood maltreatment are consistent with research indicating that reward processing is a transdiagnostic mechanism in the development of psychopathology, potentially via attenuated motivation (Whitton, Treadway, & Pizzagalli, 2015).

We found no significant group differences using our computational model‐based analysis of the brain imaging data. The partial fit of the model across the sample may have contributed to this null finding, as our sample size was reduced by approximately one‐third for this analysis. Although there is previous work suggesting that this task is valid in children and adolescents (Manning, Kilner, Neil, Karaminis, & Pellicano, 2017), it is important to note that the previous study adopting these methods in fMRI focused on a non‐clinical adult sample (Behrens et al., 2007). By employing a model comparison approach, we tried to ensure the validity of the winning‐model, but such an approach does necessitate a certain methodological rigour that can result in the exclusion of some participants (e.g. Liu, Valton, Wang, Zhu, & Roiser, 2017), that is whenever their data do not sufficiently support any of the computational models. A better fit to the null model indicates random decision making. Additional analyses showed that random decision making was not associated to maltreatment or psychopathology. This indicates that differences in model fit are not related to the measures if interest in this study, that is it is unlikely that these differences influenced the current results. The Bayesian observer model reported previously (Behrens et al., 2007) was also not sufficiently supported by the data in this sample of children and adolescents. Further computational research is needed to address reasons for individual differences in model fit.

Research on the development of both behavioural characteristics as well as neural systems underlying reinforcement learning indicates substantial developmental alterations over the course of childhood and adolescence (DePasque & Galvan, 2017), including structural changes in grey and white matter volume and functional changes in adjusting to feedback in a fronto‐thalamic network (van Duijvenvoorde, Achterberg, Braams, Peters, & Crone, 2016). While the focus of this study was on the effects of maltreatment experience, additional analyses across the entire group provided evidence that in children and adolescents from diverse backgrounds computational processes map on to the expected neural substrates (e.g. prediction errors in the striatum), consistent with prior research (e.g. Cohen et al., 2010; Van den Bos, Cohen, Kahnt, & Crone, 2012). More developmental research is needed, particularly in normative populations, to better characterise reward learning and its computational bases, also across different conditions of volatility.

Several limitations of this study merit comment. First, while our sample size was adequate to detect differences across groups, it did not support the analysis of subgroup effects (e.g. in relation to potential effects of sex). There is theoretical and empirical support for differential effects of abuse and neglect (McLaughlin, Sheridan, Humphreys, Belsky, & Ellis, 2021; Puetz et al., 2020; however see also Smith & Pollak, 2021). However, due to high rates of overlapping exposure to both abuse and neglect as well as a relatively limited sample size in terms of subgroup analyses, it was not possible to differentiate these forms of adversity in the current study. There is an ongoing discussion about what represents a sufficient sample size for neuroimaging research, especially studies examining between‐subjects associations (Marek et al., 2022; but see also DeYoung et al., 2022). Importantly, power analyses for the current study indicated good power to detect group differences in a study of *experimentally induced* brain activations. While power to detect associations with individual differences will be lower, the sample size of the current study compares favourably to other studies of children with maltreatment experience who represent a high‐risk and difficult to access population (e.g. Dennison et al., 2016; Hart et al., 2018; McLaughlin et al., 2015; Puetz et al., 2023). Nonetheless, owing to the limited sample size the associations we report should be treated with caution and require independent replication. Second, an important limitation is the poor fit to the tested computational models for approximately one third of our sample, which limited the power of our computational analyses. While the results indicated that no systematic bias was introduced by the exclusion of the respective participants, there is a need for further research into the potential reasons of poor model fit in some participants. Third, despite systematic efforts of the research team to retain as many participants as possible even during an ongoing pandemic, retained participants in the MT groups had significantly higher SES than those who had dropped out which is a limitation with regard to our sample's representativeness. Additionally, numerically more participants dropped out in the MT group, although this result narrowly fell short of statistical significance. Fourth, to further understand changes associated with maltreatment experience as potential adaptations to environmental factors, future research should employ additional measures of environmental characteristics (e.g. Glynn et al., 2019). Finally, ethical considerations do not allow a random assignment of participants to maltreatment status. It is therefore not possible to causally attribute any observed differences to maltreatment experience, although in our view this seems to represent a plausible inference.

In summary, we found that children and adolescents with maltreatment experience showed less exploratory behaviour in volatile environments, a pattern that appears protective against future internalising symptomatology, but which may incur longer term costs. Furthermore, we found that volatility of the reward environment was associated with a pattern of altered neural OFC activation and OFC‐MCC connectivity in children and young people with maltreatment experience. Altered OFC‐MCC connectivity during volatility was also associated with increased psychopathology at follow‐up. These findings provide preliminary evidence that exposure to harsh, unpredictable early learning environments may impact reward processing in ways that contribute to increased risk of psychiatric disorder. This highlights the importance of reward learning as a putative latent vulnerability mechanism that may have implications for both prevention and intervention approaches.

## Ethical approval

This study was approved by the UCL Research Ethics Committee (11767/001). The participants' parents or legal guardians provided written consent, and all participants provided written assent.

**Key points**

- Childhood maltreatment is a significant risk factor for mental health problems and social difficulties.
- Atypical reward learning across different environmental contexts has been reliably associated with several psychiatric disorders, including depression.
- Maltreatment experience was associated with lower propensity to explore across varying reward environments.
- Neural activation of the orbitofrontal cortex during reward learning was lower in those with maltreatment experience relative to peers, also showing altered connectivity to the medial cingulate cortex.
- These findings provide evidence that atypical reward learning is implicated in latent vulnerability to mental health problems in children and adolescents following maltreatment experience earlier in childhood.

## Supporting information


**Table S1.** Documented maltreatment experience, severity, estimated duration and age of onset (both shown in years).
**Table S2.** Complete breakdown of ethnicities.
**Figure S1.** Model comparisons.
**Table S3.** Comparison between those with poor model fit and those with sufficient model fit.
**Figure S2.** Prediction error related activation in the striatum across groups.
**Table S4.** Longitudinal results for externalizing symptoms.
**Table S5.** Descriptive Statistics and group comparisons for follow‐up data.
**Table S6.** Within group differences between retained and dropped out participants for MT and NMT groups.

## Data Availability

The data that support the findings of this study are available on request from the corresponding author. The data are not publicly available due to privacy or ethical restrictions.
