# Supplementary material for: Childhood maltreatment is associated with lower exploration and disrupted prefrontal activity and connectivity during reward learning in volatile environments
Source: J Child Psychol Psychiatry. 2024 Dec 12;66(6):846–56. doi: 10.1111/jcpp.14095 (PMC12062857; doi:10.1111/jcpp.14095)
Supplement: Supplementary file 1 — Table S1. Documented maltreatment experience, severity, estimated duration and age of onset (both shown in years). Table S2. Complete breakdown of ethnicities. Figure S1. Model comparisons. Table S3. Comparison between those with poor model fit and those with sufficient model fit. Figure S2. Prediction error related activation in the striatum across groups. Table S4. Longitudinal results for externalizing symptoms. Table S5. Descriptive Statistics and group comparisons for follow‐up data. Table S6. Within group differences between retained and dropped out participants for MT and NMT groups. [file JCPP-66-846-s001.docx]

**Supporting Information** for *“Childhood maltreatment is associated with lower exploration and disrupted prefrontal activity and connectivity during reward learning in volatile environments”*

**Supplemental Methods**

*Maltreatment history*

Table S1. Documented maltreatment experience, severity, estimated duration and age of onset (both shown in years).

|  |  | **N** | **Mean** | **SD** |
| --- | --- | --- | --- | --- |
| **Neglect** | Severity (0-4) | 27 | 2.37 | 1.63 |
|  | Duration |  | 5.74 | 4.22 |
|  | Age of onset |  | 2.37 | 2.97 |
| **Sexual abuse** | Severity (0-4) | 2 | 0.14 | 0.69 |
|  | Duration |  | 1.04 | 1.36 |
|  | Age of onset |  | 6.96 | 4.23 |
| **Emotional abuse** | Severity (0-4) | 34 | 2.60 | 1.19 |
|  | Duration |  | 7.38 | 4.87 |
|  | Age of onset |  | 4.68 | 4.23 |
| **Home violence** | Severity (0-4) | 14 | 0.49 | 0.73 |
|  | Duration |  | 4.57 | 3.20 |
|  | Age of onset |  | 4.27 | 2.46 |

Senior social work professionals double-rated six (16%) randomly selected cases: interrater reliabilities showed 100% agreement in relation to the presence of sexual abuse, 92.8% for neglect and 85.7% for home violence and emotional abuse.

Table S2. Complete breakdown of ethnicities.

|  | Maltreatment Group (N=37) | Non-maltreatment Group (N=32) |
| --- | --- | --- |
| White British/White Irish/White Other, n [%] | 16 [43] | 15 [47] |
| Black British/Black African/Black Caribbean/ Black Other, n [%] | 8 [22] | 7 [22] |
| Asian British/Asian Other, n [%] | 2 [5] | 1 [3] |
| Multiple ethnic groups, n [%] | 10 [27] | 7 [22] |
| Other, n [%] | 1 [3] | 2 [6] |

For those participants who were followed up after the start of the Covid-19 pandemic, carers also completed the Coronavirus Health Impact Survey (CRISIS) ‘Baseline Current Form’ (https://github.com/nimh-comppsych/CRISIS) assessing the impact of the pandemic on the child from which an emotional impact subscale was calculated. Results can be found in Table S3.

*Computational Analyses*

A computational modelling approach was used to analyse the participants’ decisions during the task on a trial-by-trial basis. This analysis allowed us to extract precise parameters governing exploration/exploitation (i.e., temperature), as well as the speed at which individuals were accumulating information (i.e., learning rate) during different stages of the task (stable vs. volatile blocks). Participants’ data was fit to a series of twelve reinforcement learning models with increasing complexity (Fig. SI 1).

We started testing simple assumptions, such as whether participants performed randomly (Null model), simply updated behaviour based on immediate feedback (“win-stay/loose-shift”), and iteratively improved the models until we could capture the participants’ decision patterns as accurately and parsimoniously as possible. All models were implemented using hierarchical Bayesian estimation in the probabilistic modelling language Stan^1,2^, allowing us to recover parameter estimates accurately^3^. Generatively, this modelling approach describes that participants are expected to come from a common group-level distribution, such that participants’ parameters are then expected to be similar to one another. Model parameters were estimated using Hamiltonian Markov Chain Monte Carlo (HMC) sampling as it allows to estimate parameters both at the group level and individual subject level simultaneously.

The Widely Applicable Information Criterion (WAIC) scale and K-fold cross validation (K-fold CV) were used to compare model fits^4,5^. The WAIC is akin to cross-validation, or other approximations such as the Akaike Information Criterion, but the WAIC is more sensitive, particularly in hierarchical modelling settings^6^. K-fold CV was used when diagnostic measures of the WAIC (pareto-k) suggested that the WAIC approximation was sub-optimal. While K-fold CV provides true cross validation metrics as opposed to the approximation yielded by the WAIC, it requires refitting each model K times with different sub-partitions of the data, and as such is substantially more computationally expensive to compute than WAIC. A lower K-fold CV or WAIC for each model indicates a better model fit, and the relative difference between the winning model and another model can be used to establish the relative strength of the evidence for one model over another. This is akin to Bayes factors^7^. We defined a difference in model evidence (𝚫WAIC) of 0-2 as weak evidence; 2-6 positive evidence; 6-10: strong evidence; >10: very strong evidence in favour of the winning model^7^.

For each model, two chains were produced with 1000 warm-up iterations and 4000 post warm-up iterations per chain. Model convergence was ensured through careful analysis of traceplots and monitoring of the Gelman-Rubin statistic (all potential scale reduction factors: R-hat < 1.1)^8,9^.

Recommendations for weakly informative priors in hierarchical logistic regression were used^1^. Following recent recommendations^10^ (Gabry et al., 2017), prior predictive calibrations were performed on the winning model family to ensure that the prior choices led to adequate decision profiles. Parameter recovery on synthetic data was carried out to ensure that all parameters were identifiable and could be adequately recovered, and finally posterior predictive checks were performed to ascertain that the winning model could capture the behavioural choice pattern of all participants. For all models, subject-level individual parameters (for the learning rates, and the initial Q-values) were Beta distributed to enforce a proportion of level between zero and one, while the temperature parameter were gamma distributed to enforce support between zero to infinity.

A total of twelve models were created:

- Model 1 : Null model (serving as benchmark)
- Model 2: Win-stay Loose-Shift
- Model 3: Simple RL (1 Temperature, 1 Learning rate)
- Model 4: RL split learning rates (1 Temperature, 1 Learning rate stable env., 1 learning rate volatile env.)
- Model 5: Two Model 3 (one RL model per environment: 1 Temp + 1 LR per environment)
- Model 6: Model 3 + initial Q-value bias
- Model 7: Model 4 + initial Q-value bias
- Model 8: Model 5 + initial Q-value bias
- Model 9: RL reward/punishment (1 Temperature, 1 learning rate from rewards, 1 learning rate for punishments)
- Model 10: Model 9 + initial Q-value bias
- Model 11: Two Model 9 (one per environment)
- Model 12: Model 11 + initial Q-value bias

Before comparing models across groups, we first we performed model comparison at the participant level, comparing each successive model against the Null model. This enabled to highlight participants that did not appear to be sufficiently well captured by the 12 modelling strategies implemented (35% of the participants demonstrated better fit by the Null model). These participants were removed from further computational analysis, as including them would have heavily skewed the model comparison results at the group level towards a simpler model than warranted (given that group model comparison is an equal-weighted sum of participants model evidence), and the estimated parameters for these users would have been uninterpretable due to their poor model fit.

We then performed model comparison at the group level (MT vs NMT) to extract the best, yet most parsimonious model for each group. NMTs were best described by a simple RL model with a single learning rate (across stable and volatile blocks), and a single temperature parameter. MTs on the other hand, were better described by a model including a starting initial bias parameter (Q0), a temperature parameter (Tau), and two learning rates (one for the stable, and one for the volatile environment – alpha_stable, alpha_vol respectively). Given that we wanted to compare the participants’ parameters across groups, we opted to extract parameters for the same model across both groups (model 7: Q0 + Tau + alpha_stable + alpha_volatile). This is because although model comparison favours a slightly simpler model for the NMT group due to having fewer free parameters, the models can be made mathematically equivalent since model 7 is nested within the simpler RL model. That is, participants that do not need the additional parameters present in model 7 (Q0, alpha_stable and alpha_volatile) can be expressed into the simpler RL model by having a Q0 of 0.5 (i.e. no initial preference for either stimuli), and having the same learning rate for volatile and stable environments (equivalent to having a single learning rate throughout the experiment).

Figure S1. Model comparisons.


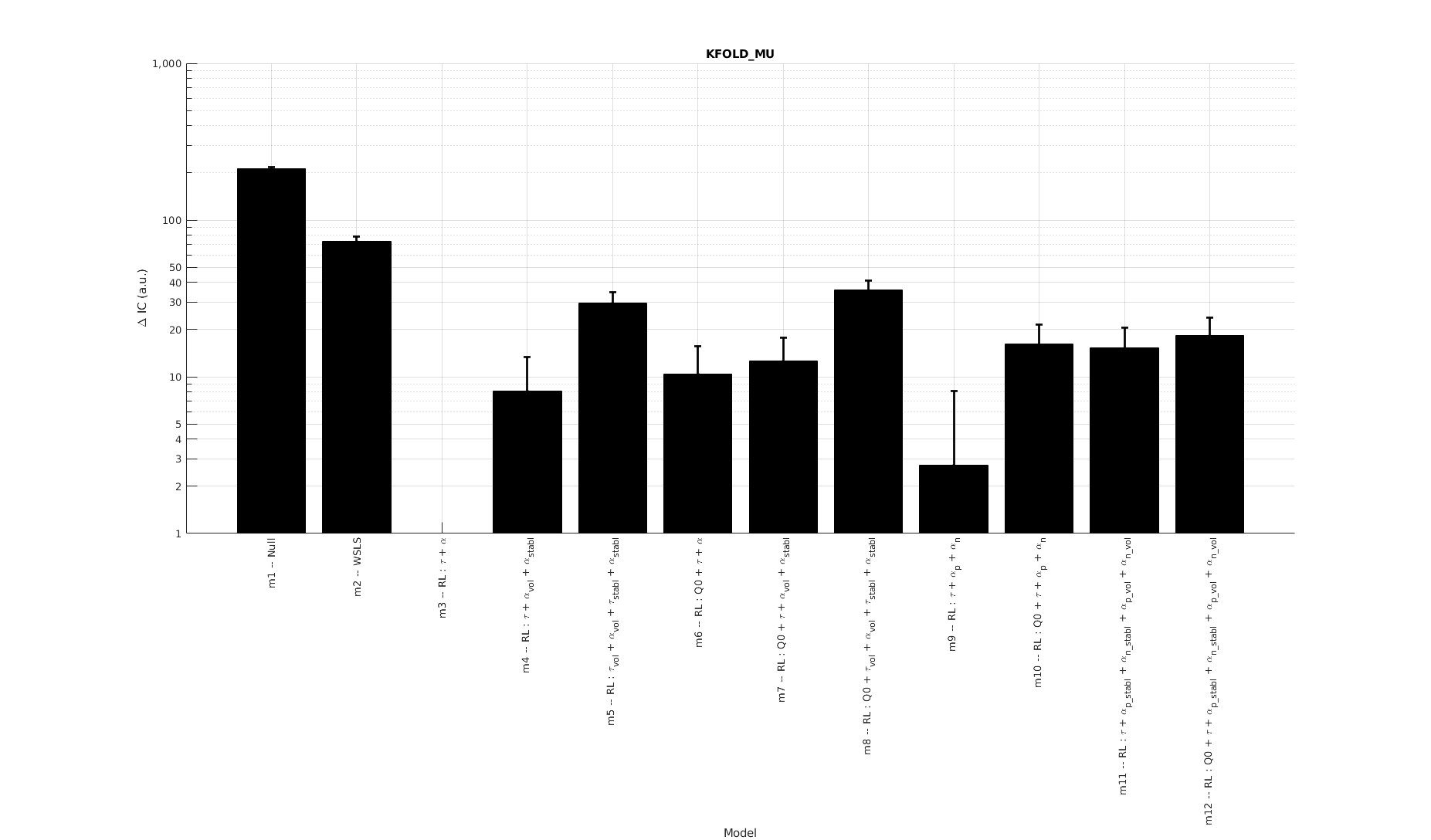


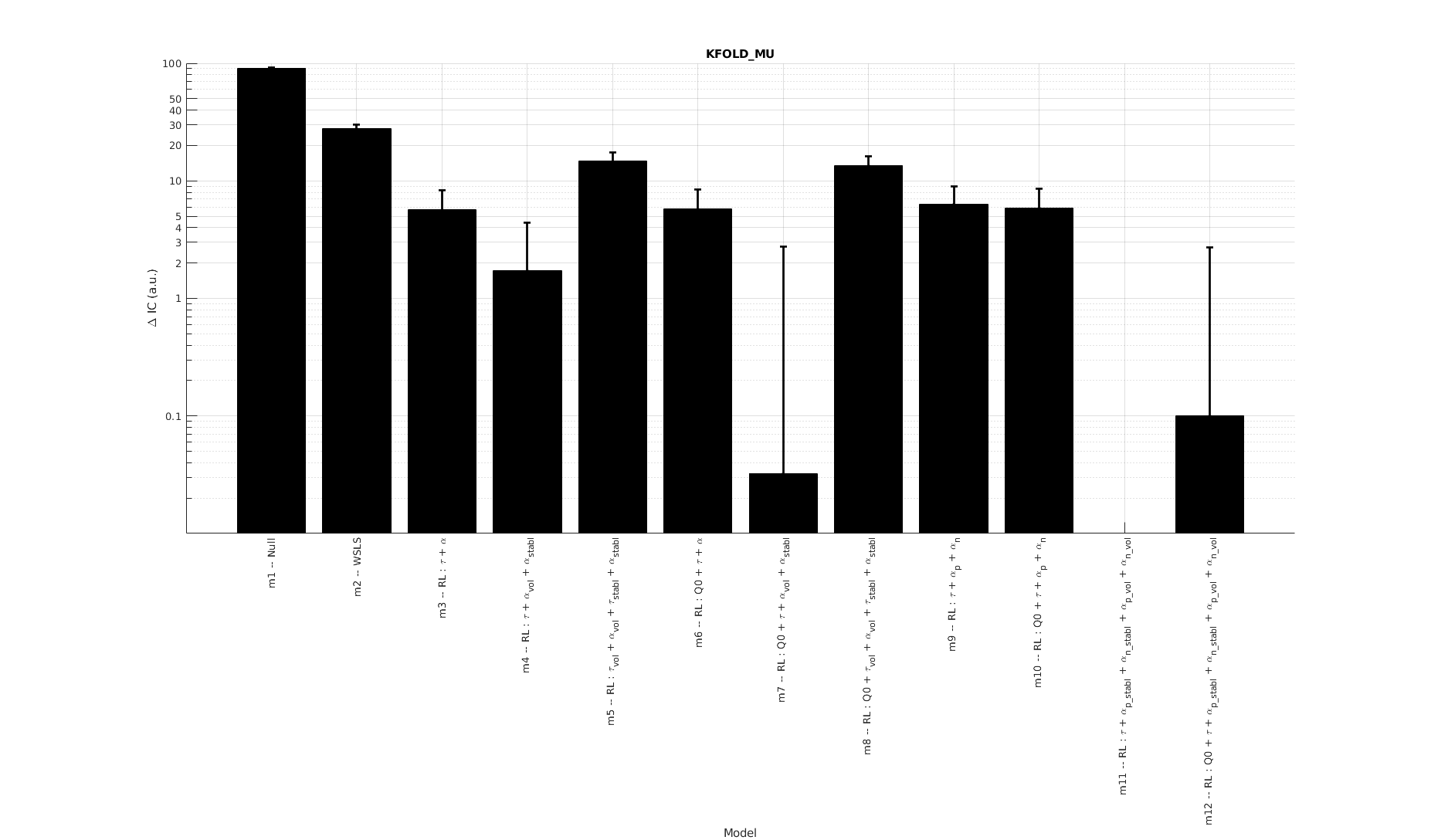


Table S3. Comparison between those with poor model fit and those with sufficient model fit.

|  | **Across groups** | | | **MT group** | | | **NMT group** | | |
| --- | --- | --- | --- | --- | --- | --- | --- | --- | --- |
|  | poor model fit  N=23 | sufficient model fit  N=46 | *p* | poor model fit  N=13 | sufficient model fit  N=24 | *p* | poor model fit  N=10 | sufficient model fit  N = 22 | *p* |
| CTQ | 14.72 (8.0) | 15.89 (8.3) | .620 | 17.60 (9.8) | 19.56 (11.6) | .662 | 11.13 (2.4) | 13.23 (2.6) | .055 |
| internalizing symptoms | 1.87 (2.3) | 2.38 (1.9) | .340 | 2.77 (2.7) | 2.83 (2.0) | .935 | 0.70 (0.7) | 1.86  (1.8) | .014* |
| general symptomatology | 9.48 (6.9) | 8.93 (5.9) | .735 | 12.54 (7.6) | 10.54 (6.4) | .401 | 5.50 (2.7) | 7.09  (4.9) | .345 |
| IQ | 104.57 (8.4) | 99.65 (11.3) | .070 | 103.23 (8.5) | 99.21 (13.1) | .327 | 106.30 (8.4) | 100.14 (9.3) | .084 |
| age | 14.14 (2.1) | 13.30 (2.0) | .118 | 14.07 (2.0) | 13.45 (2.1) | .378 | 14.22 (2.4) | 13.15 (2.0) | .200 |
| sex [female/male] | 11/12 | 26/20 | .495 | 6/7 | 10/14 | .478 | 5/5 | 10/12 | .811 |
| ethnicity [white/non-white] | 14/9 | 17/29 | .060 | 8/5 | 8/16 | .098 | 6/4 | 9/13 | .316 |
| SES | 3.26 (0.8) | 3.28 (0.9) | .829 | 3.38 (0.9) | 3.31 (1.1) | .863 | 3.10 (0.7) | 3.25 (0.8) | .589 |

*FMRI analyses*

*FMRI data acquisition.* Participants were scanned on a 1.5 Tesla Siemens Avanto MRI scanner (Siemens Medical Systems, Erlangen, Germany) using a 32-channel head coil and whole-brain multiband echo-planar imaging (EPI) sequence (multiband acceleration factor: 3, TR: 1300ms; TE: 54.3 milliseconds; voxel size: 3 x 3 x 3 mm; slices per volume, 39; slice thickness: 3mm; field of view: 192 mm; flip angle: 62 degree). Most participants completed one task run lasting approximately 23 min resulting in 910 EPI volumes on average; for some participants this was divided across 2 runs with the break never happening around the switch from one experimental phase to the other. A magnetization-prepared rapid gradient-echo sequence (MP-RAGE) was used to obtain a high-resolution anatomical scan (parameters: 176 slices; slice thickness: 1 mm; gap between slices: 0.5 mm; TE: 2730 milliseconds; TR: 3.57 milliseconds; voxel size: 1 x 1 x 1 mm; field of view: 256 mm).

*FMRI preprocessing.* After discarding the first eight volumes to allow for magnetic equilibration, volumes were realigned to the first image and unwarped using SPM12’s default options. Next, the anatomical scan was co-registered to the mean functional image. Using the deformation parameters of the anatomical scan’s segmentation at a 3x3x3 voxel size, functional images were normalized into Montreal Neurological Institute (MNI) standard space, smoothed using an 8 mm Gaussian kernel.

Frame-to-frame displacement was assessed and images with movements of >1.5 mm or >0.5 degrees rotation were replaced by interpolations of adjacent images where corruption of the scan was detected. The groups did not differ significantly on average absolute movement (p > .4).

*Subject-level analysis.* Each individual’s timeseries was analysed using a multiple regression model including the six motion regressors as well as one regressor for each corrupted scan (identified by the above-described procedure) in order to reduce motion related artifacts. To further reduce any effects of physiological artifacts, timeseries from white matter and cerebrospinal fluid regions were included. These were extracted using the TAPAS toolbox implemented in CONN^11^ using the first five principal components at a threshold of 0.9^12,13^. Task related activation was modelled using fixed-effects regressors through convolution with SPM’s canonical hemodynamic response function in two ways: 1) in a computational model-informed analysis, using the individual trial-by-trial estimates of expected value derived from the model as a parametric modulator in the decision phase, and trial-by-trial estimates of prediction error in the outcome phase (in a reduced sample showing adequate model fit statistics: N=24 MT, N=22 NMT); 2) in a standard model-agnostic analysis, incorporating regressors corresponding to win and loss outcomes in the stable and volatile conditions, in both the decision phase as well as in the outcome phase; their interactions were also included (in the full sample).

*Second-level analysis.* Whole brain results were analysed applying a stringent correction for multiple comparisons using AFNI’s latest 3dttest++ in combination with the 3dClustSim routine (‘-Clustsim’ option) (https://afni.nimh.nih.gov/pub/dist/doc/program_help/ 3dClustSim.html), which uses permutation testing to derive cluster size thresholds corresponding to p < .05 FWE corrected.

**Supplemental Results**

Figure S2. Prediction error related activation in the striatum across groups.

**
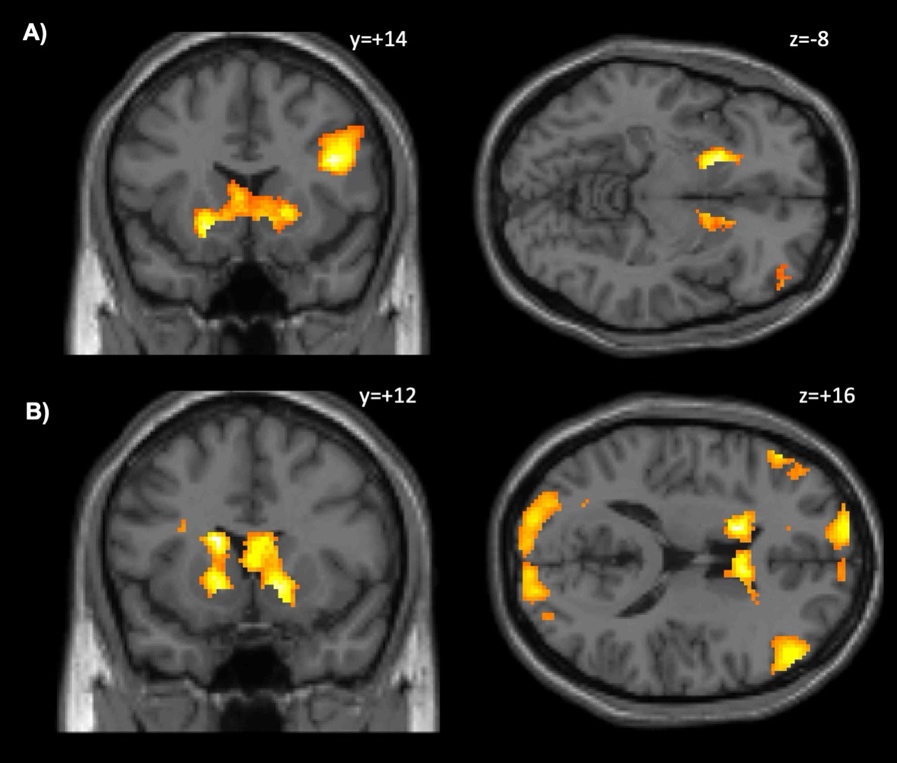
**

*Note.* Activation across groups related to A) prediction errors in the stable phase, B) prediction errors in the volatile phase.

*Exploratory results for externalising symptoms*

Exploratory analyses did not identify significant associations between OFC activation and externalizing symptoms, i.e., SDQ subscales conduct problems (MT: *r*=.06, *p*=.706; NMT: *r*=-.08, *p*=.665) and hyperactivity (MT: *r*=.08, *p*=.633; NMT: *r*=-.26, *p*=.166).

Table S4 shows the results of longitudinal analyses regarding externalizing symptoms, i.e., SDQ subscales conduct problems and hyperactivity.

Table S4. Longitudinal results for externalizing symptoms.

| Prediction by Behavioural temperature | | | | |  | | |
| --- | --- | --- | --- | --- | --- | --- | --- |
|  | | | | | *total model* | | |
|  | *ß* interaction  (group x behavioural scale) | *t* | *p* | *r^2^_part_* | *R^2^* | *F* | *p* |
| conduct problems | .172 | .77 | .432 | .105 | .352 | 3.51 | .006 |
| hyperactivity | .273 | 1.21 | .238 | .168 | .302 | 2.95 | .016 |
| Prediction by OFC activation | | | | |  | | |
|  | | | | | *total model* | | |
|  | *ß* interaction  (group x behavioural scale) | *t* | *p* | *r^2^_part_* | *R^2^* | *F* | *p* |
| conduct problems | -.043 | -.34 | .735 | -.034 | .451 | 6.65 | <.001 |
| hyperactivity | -.201 | -1.67 | .102 | -.164 | .479 | 7.21 | <.001 |
| Prediction by OFC-MCC connectivity | | | | |  | | |
|  | | | | | *total model* | | |
|  | *ß* interaction  (group x behavioural scale) | *t* | *p* | *r^2^_part_* | *R^2^* | *F* | *p* |
| conduct problems | .143 | .920 | .363 | .093 | .440 | 6.40 | <.001 |
| hyperactivity | .274 | 1.81 | .077 | .176 | .487 | 7.41 | <.001 |

*Effects of Covid-19 pandemic*

Until the first lockdown in England due to the Covid-19 pandemic 6 MT participants out of a total of 27 and 6 NMT participants out of a total of 31 had completed the follow-up. After a transition period, we started to collect data on the potential impact of the pandemic on participants’ emotional well-being. After obtaining the respective ethics committee’s decision we used the Coronavirus Health Impact Survey (CRISIS) ‘Baseline Current Form’ (<https://github.com/nimh-comppsych/CRISIS>) and an emotional impact subscale was calculated from items 3, 4, 5, 6, 16, 18, 19, 21 and 22 which showed good internal consistency (Cronbach’s alpha = 0.84). Results showed that according to these measures at the time of follow-up, the emotional impact of the pandemic had been similar for MT and NMT participants (Table S5). There were also no significant differences between groups in the number of significant life events reported on the Coddington scale (Table S5).

Table S5. Descriptive Statistics and group comparisons for follow-up data.

|  | **MT**  **Mean (SD)** | **NMT**  **Mean (SD)** | ***p*** |
| --- | --- | --- | --- |
| Follow-up interval (days) *(SD)* | 574.0 *(30.3)* [N=27] | 573.1 *(28.8)*  [N=29] | .915 |
| Subscale from CRISIS ‘Emotional Impact of Pandemic’ | 15.5 (7.0)  [N=12] | 15.5 (5.1)  [N=21] | .717 |
| Number of participants reporting negative life-events in Coddington | 4 | 2 | .503 |

Table S6. Within group differences between retained and dropped out participants for MT and NMT groups.

| **Measure** | **MT group** | | ***p*** | **NMT group** | | ***p*** |
| --- | --- | --- | --- | --- | --- | --- |
|  | **retained** | **dropped-out** |  | **retained** | **dropped-out** |  |
| maltreatment severity [CTQ Total] | 19.55 | 16.33 | .534 | 12.57 | 14.00 | .474 |
| baseline internalizing symptomatology | 3.11 | 2.00 | .183 | 1.54 | 1.00 | .588 |
| baseline general symptomatology | 10.93 | 12.1 | .647 | 6.71 | 5.33 | .607 |
| age | 13.54 | 14.01 | .543 | 13.68 | 11.56 | .109 |
| sex [female/male] | 16/11 | 4/6 | .297 | 16/13 | 1/2 | .471 |
| IQ | 101.67 | 97.80 | .381 | 102.38 | 99.00 | .560 |
| pubertal status | 2.59 | 2.72 | .668 | 2.41 | 1.75 | .218 |
| SES | 3.10 | 4.10 | .005^**^ | 3.14 | 3.83 | .185 |

*Note.* ^**^p<.01. Higher values in the SES measure indicate lower SES.

**Supplemental Reference List**

1. Gelman, A. & Hill, J. *Data Analysis Using Regression and Multilevel/Hierarchical Models*. (Cambridge University Press, Cambridge ; New York, 2007).

2. Carpenter, B. *et al.* Stan: A Probabilistic Programming Language. *J Stat Softw* **76**, 1–29 (2017).

3. Valton, V., Wise, T. & Robinson, O. J. Recommendations for Bayesian hierarchical model specifications for case-control studies in mental health. NeurIPS, Machine Learning for Health (ML4H). Preprint at http://arxiv.org/abs/2011.01725 (2020).

4. Watanabe, S. A Widely Applicable Bayesian Information Criterion. *J Mach Learn Res* **14**, 867–897 (2013).

5. Vehtari, A., Gelman, A. & Gabry, J. Practical Bayesian model evaluation using leave-one-out cross-validation and WAIC. *Stat Comput* **27**, 1413–1432 (2017).

6. Gelman, A., Hwang, J. & Vehtari, A. Understanding predictive information criteria for Bayesian models. (2013).

7. Kass, R. E. & Raftery, A. E. Bayes Factors. *Journal of the American Statistical Association* **90**, 773–795 (2012).

8. Brooks, S. P. & Gelman, A. General Methods for Monitoring Convergence of Iterative Simulations. *Journal of Computational and Graphical Statistics* **7**, 434–455 (2012).

9. Gelman, A. & Rubin, D. B. Inference from Iterative Simulation Using Multiple Sequences. *Statistical Science* **7**, 457–472 (1992).

10. Gabry, J., Simpson, D., Vehtari, A., Betancourt, M. & Gelman, A. Visualization in Bayesian workflow. in *arXiv.org* (John Wiley & Sons, Ltd, 2017).

11. Whitfield-Gabrieli, S. & Nieto-Castanon, A. Conn: a functional connectivity toolbox for correlated and anticorrelated brain networks. *Brain Connect* **2**, 125–41 (2012).

12. Behzadi, Y., Restom, K., Liau, J. & Liu, T. T. A component based noise correction method (CompCor) for BOLD and perfusion based fMRI. *Neuroimage* **37**, 90–101 (2007).

13. Li, Y., Saxe, R. & Anzellotti, S. Intersubject MVPD: Empirical comparison of fMRI denoising methods for connectivity analysis. *PloS one* **14**, e0222914 (2019).
